# Interactions among the *A* and *T* Units of an ECF-Type Biotin Transporter Analyzed by Site-Specific Crosslinking

**DOI:** 10.1371/journal.pone.0029087

**Published:** 2011-12-27

**Authors:** Olivia Neubauer, Christin Reiffler, Laura Behrendt, Thomas Eitinger

**Affiliations:** Institut für Biologie/Mikrobiologie, Humboldt-Universität zu Berlin, Berlin, Germany; Swiss Federal Institute of Technology Zurich, Switzerland

## Abstract

Energy-coupling factor (ECF) transporters are a huge group of micronutrient importers in prokaryotes. They are composed of a substrate-specific transmembrane protein (S component) and a module consisting of a moderately conserved transmembrane protein (T component) and two ABC ATPase domains (A components). Modules of A and T units may be dedicated to a specific S component or shared by many different S units in an organism. The mode of subunit interactions in ECF transporters is largely unknown. BioMNY, the focus of the present study, is a biotin transporter with a dedicated AT module. It consists of the S unit BioY, the A unit BioM and the T unit BioN. Like all T units, BioN contains two three-amino-acid signatures with a central Arg residue in a cytoplasmic helical region. Our previous work had demonstrated a central role of the two motifs in T units for stability and function of BioMNY and other ECF transporters. Here we show by site-specific crosslinking of pairs of mono-cysteine variants that the Ala-Arg-Ser and Ala-Arg-Gly signatures in BioN are coupling sites to the BioM ATPases. Analysis of 64 BioN-BioM pairs uncovered interactions of both signatures predominantly with a segment of ∼13 amino acid residues C-terminal of the Q loop of BioM. Our results further demonstrate that portions of all BioN variants with single Cys residues in the two signatures are crosslinked to homodimers. This finding may point to a dimeric architecture of the T unit in BioMNY complexes.

## Introduction

ECF-type ABC transporters are widespread among prokaryotes and involved in uptake of vitamins, transition metal cations, intermediates of salvage pathways and probably other compounds [1]. Research on these systems originated during work on import systems for cobalt and nickel ions [2] and for the vitamin biotin [3], [4], [5]. The analyses uncovered that two subunits, an NBD (called A component) and a conserved transmembrane protein (T component), of the metal transporters and the biotin transporters, are related. Functional genomics then uncovered many more transporters of this type. The description in 2009 [6] of ECF systems as a novel group of membrane transporters for many different substrates contradicted the dogma that ABC-type importers strictly depend on extracytoplasmic soluble solute-binding proteins for delivery of substrate and initiation of the transport cycle. Instead, ECF importers contain substrate-specific (“S”) transmembrane proteins. S units are in most cases single small (20–25 kDa) membrane proteins and have extremely high affinity for their substrates in the low nanomolar or picomolar range [7], [8], [9]. The primary structures of the S components for different substrates are highly diverse. T components are moderately similar transmembrane proteins with strongly conserved amino acid signatures in a cytoplasmic loop. Since the A components contain the typical features of NBDs including the Walker A and B motifs, the LSGGQ signature sequence and the His motif, they are predicted to function as dimers as all ABC ATPases. The module composed of A and T units is called – for historical reasons – the “energy-coupling factor” (ECF).

Another unprecedented finding was the fact that the ECF module is shared by several highly diverse S components in one subgroup of ECF transporters (called subgroup II) which are mainly found among gram-positive bacteria and archaea. Subgroup I comprises systems with a dedicated ECF module in gram-negative and gram-positive bacteria and in archaea. Notably, the S components of two bacterial cobalt transporters and the biotin transporter BioMNY of *Rhodobacter capsulatus*, which are members of subgroup I, were shown by in vivo assays to have significant substrate-uptake activity in the absence of their cognate A- and T units [2], [5], [10], [11]. In contrast, analysis of vitamin uptake by subgroup II folate, pantothenate, riboflavin and thiamine transporters suggest that the corresponding S components FolT, PanT, RibU and ThiT do not function as transporters in a solitary state [6], [12], [13], [14].

Many questions regarding physical and functional interactions among the subunits of ECF transporters and their in vivo oligomeric state remain to be answered. Furthermore, the role of the T components is still not understood. Light-scattering experiments with purified subgroup II ECF transporters of *L. lactis* have revealed that the S, A1, A2 and T subunits mainly exist in a 1∶1∶1∶1 stoichiometry in detergent solution [15]. On the other hand, in vivo fluorescence analyses of the subgroup I biotin transporter (BioMNY) of *R. capsulatus* suggest, that the S unit BioY oligomerizes in the living cell independent of the presence of the A (dimer of BioM) and T (BioN) components [11]. This finding is indicative of a transporter complex with a higher-order structure in situ.

The T components of ECF transporters may function as docking sites for the membrane-spanning S units and the cytoplasmic A units. Recent crystal structure analysis of the *L. lactis* S unit ThiT combined with sequence comparisons and mutant studies suggest that an Ala-X_3_-Ala (where X is any amino acid) signature in transmembrane helix I of the *L. lactis* S units is involved in S unit:T unit interactions [14]. Two signatures with Ala-Arg-Gly as the consensus in a cytoplasmic loop with predicted helical structure are the most conserved feature among all T components and may be responsible for T unit:A unit interactions [1]. Individual replacements of the two Arg residues were shown to inactivate and destabilize subgroup II ECF transporters and to reduce ATPase activity of the *R. capsulatus* BioMNY biotin transporter. A double replacement in BioN abolished complex formation completely [12]. These findings led us to hypothesize that the cytoplasmic Arg-containing motifs of T components could be contact sites for physical and functional interaction with the cytoplasmic A units [12]. This scenario would resemble in part the organization of classical ABC transporters in which coupling helices of the transmembrane domains interact with a groove in the NBDs formed by residues in and around the Q loop (reviewed in refs. [16], [17], [18], [19]).

In the present study, we chose the BioMNY system and analyzed the potential physical interaction of the two Arg-containing (ARS and ARG) motifs in the T unit BioN with the A unit BioM by site-specific crosslinking. Nine mono-cysteine variants of BioN with single Cys residues in and around the ARS and ARG sequences were constructed and co-produced with BioM variants containing single Cys residues in the Q loop and the adjacent helical domain. Among 64 combinations analyzed, 28 gave distinct and pronounced thiol crosslinking products. This indicates that the ARS/ARG-containing region of the T unit and the region adjacent to the Q loop of the A units indeed are in physical contact. Moreover, our observation that all nine mono-Cys BioN variants are partially crosslinked to give homodimers may indicate an oligomeric arrangement of the T component in BioMNY complexes.

## Results

### Biotin transporter complexes containing c-Myc-tagged BioN

**Figure 1:**
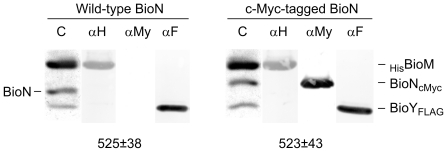
Subunit composition of biotin transporter complexes with wild-type and c-Myc-tagged BioN. Protein complexes were purified by nickel-chelate affinity chromatography via the His-tag on BioM, subjected to SDS PAGE and blotted onto nitrocellulose membranes. The membranes were processed with anti-oligo-His (*αH*), anti-c-Myc-tag (*αMy*) or anti-FLAG (*αF*) antibody alkaline phosphatase conjugates. *C* indicates Coomassie blue-stained lanes of the gels. The values in the lower part give the mean ATPase activity ± the standard deviation in nmol inorganic phosphate produced from ATP per min and per mg of protein for BioMNY with wild-type (18 assays) and cMyc-tag-containing (10 assays) BioN.

In the natural host, the *bioN* and *bioY* genes in the *bioMNY* operon overlap by 11 nucleotides (including the *bioN* stop codon) and a TTG triplet serves as the intiation codon of *bioY*. To increase the expression efficiency, the TTG had previously been replaced by an ATG codon in a plasmid encoding N-terminally deca-His-tagged BioM, untagged BioN and C-terminally FLAG-tagged BioY [5]. In the present study, we fused a c-Myc tag to the C-terminus of BioN allowing immunological detection of all three components of the biotin transporter via their individual tags. By this construction, the terminal three codons of *bioN* were replaced by the c-Myc-tag-encoding sequence. The TGA termination codon of the modified *bioN* and the ATG initiation codon of *bioY* overlap by the A nucleotide (Fig. S1 in the Supplement). In order to exclude the possibility that the sequence manipulation led to reduced expression levels of *bioY*, biotin transporter complexes with and without the c-Myc-tag on BioN were purified via the His-tag on BioM (yielding approx. 2 mg of protein per 1 liter of cell suspension for the two variants) and subjected to SDS PAGE and subsequent immunoblotting. As shown in Fig. 1, the relative amounts of the three peptides were unaltered and the two types of BioMNY complexes had comparable ATPase activity. ATPase activity is superior to biotin-transport activity as an indicator of BioMNY complex integrity because BioY mediates low-affinity biotin uptake even in the absence of BioMN [5]. As expected, the tag on BioN led to a slightly reduced electrophoretic mobility and allowed the detection of this peptide with the anti-c-Myc-tag antibody.

### Properties of cysteine-free BioMNY

**Figure 2:**
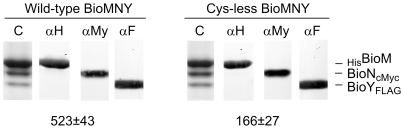
Comparison of purified wild-type and Cys-less BioMNY complexes. Samples were analyzed by SDS PAGE, Western blotting and ATPase activity assays. The procedures and the abbreviations are explained in the legend to Figure 1. The values in the lower part give the ATPase activity for triple-tagged wild-type (means of 10 assays) and Cys-less (means of 8 assays) ± the standard deviation.

As a prerequisite for studying interactions among BioM and BioN peptides in BioMNY complexes by site-specific thiol crosslinking, the natural cysteine residues in BioM (C133, C145, C235) and BioN (C8) were removed. Wild-type and quadruple-mutant complexes were present in comparable amounts in recombinant *E. coli* cells and similar quantities of the two variants could be purified. As illustrated in Fig. 2, there was no indication of reduced stability as a consequence of the cysteine replacements. Nevertheless, ATPase activity of the mutant complexes was significantly diminished. As indicated in Fig. 2, the mutant complexes had approx. one third of ATPase activity compared to the wild type. This effect could not be assigned to replacement of a specific Cys residue in BioM or BioN, but rather was an additive consequence of the four replacements. Mutant forms containing single Cys-to-Ala replacements or the ΔA_234_C_235_ deletion were only slightly affected compared to double and triple mutant forms which showed more severely reduced ATPase activity (data not shown). In spite of the reduced ATPase activity, the quadruple mutant was considered suitable for the crosslinking study.

### Mono-cysteine variants of BioM

Since the Q loop and the adjacent helical domain of the NBDs represent an interaction site with the TMDs in canonical ABC transporters, we hypothesized that similar interactions may occur in ECF importers. To identify such potential interaction sites by cysteine crosslinking, a set of 13 BioM variants with single cysteine residues in the Q loop region (by exchange of F82 or D86) and the adjacent helical domain (by exchange of I90, V94, I98, L102, G106, A110, A114, A118, A122, A126 or D130) was constructed. With the exception of the BioMNY complexes with the F82C and I90C replacements in BioM, which had only background ATPase activity, the remaining 11 mono-cysteine variants plus the variant containing the natural C133 as the solitary cysteine residue could be purified in similar quantities from recombinant *E. coli* cells and most of them showed comparable ATPase activity relative to the cysteine-free form. The D86C and A110C mutations correlated reproducibly with increased ATPase activity (Fig. S2 in the Supplement). The F82C and I90C forms were discarded and twelve variants were used for further experiments.

**Figure 3:**
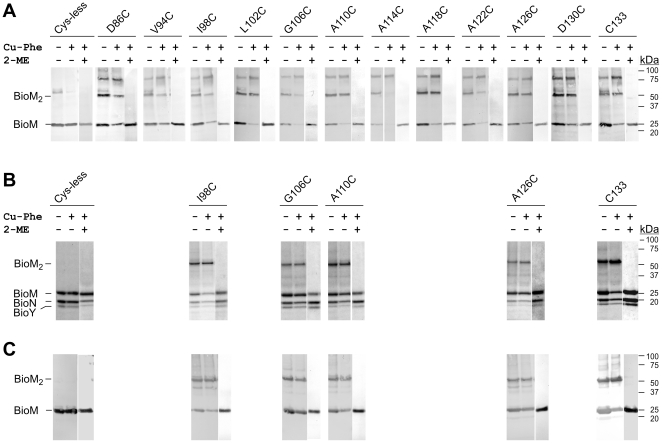
Crosslinking of His-tagged mono-cysteine BioM variants in BioMNY complexes. *A.* Crosslinking with isolated membranes containing BioMNY complexes with a mono-Cys BioM as indicated plus untagged BioN and FLAG-tagged BioY. Upon crosslinking, membranes were solubilized with SDS-containing sample buffer, the protein mixture was separated by SDS PAGE and blotted onto a nitrocellulose membrane, and the membrane was processed with anti-oligo-His-alkaline phosphatase antibody conjugates. *B.* Coomassie blue-stained gel strips. Purified BioMNY complexes in detergent solution were crosslinked and subjected to SDS PAGE. *C.* Westernblot with anti-oligo-His antibodies of the samples shown in *B*. *Cu-Phe*, copper-phenanthroline; *2-ME*, 2-mercaptoethanol.

To address the question whether thiol-crosslinked BioM dimers were formed, isolated membranes containing BioMNY complexes with mono-cysteine BioM variants were treated with the oxidizing agent Cu-phenanthroline (Cu-Phe), and subsequently solubilized with SDS and analyzed by SDS PAGE and Western blotting. The results are shown in Fig. 3A. Tagged BioM has a molecular mass of 26.8 kDa. In the absence of 2-mercaptoethanol (2-ME) as a reductant (−Cu-Phe/−2-ME; +Cu-Phe/−2-ME), two additional species with molecular masses of ∼50 kDa and ∼80 kDa were detected by the anti-oligo-His antibody in the case of the mono-Cys variants. Another somewhat diffuse band comigrated with about ∼55 kDa in most of the −Cu-Phe/−2-ME lanes including the lane with the Cys-less BioMNY-containing membranes. In the presence of reductant (+Cu-Phe/+2-ME), only the monomeric BioM was visible in each case. We interpreted these findings as follows: The ∼50 kDa species of all mono-Cys BioM mutant proteins represents BioM_2_ which is built in the absence of Cu-phenanthroline by spontaneous oxidation under air, and in the presence of Cu-phenanthroline. BioM_2_ cannot be formed by the Cys-less BioM. The ∼80 kDa species is an oxidation product of the mono-Cys BioM proteins with an unrelated protein contained in the membrane preparations. This oxidized form does not occur in the case of the Cys-less BioM. The ∼55 kDa species may represent an aggregate of crosslinked membrane proteins unrelated to BioMNY that is detected by the anti-oligo-His antibody. Addition of Cu-phenanthroline may cause formation of superaggregates that do not migrate in the gel, and addition of reductant may resolve the aggregates to give a number of low-molecular-weight peptides.

To confirm our assumption that the ∼50 kDa species represents crosslinked BioM ( = BioM_2_), some of the BioMNY complexes with single cysteine residues in BioM were purified via the His-tag on BioM and subjected to crosslinking in detergent solution. Fig. 3B and Fig. 3C illustrate the results. Both Coomassie staining after SDS PAGE (Fig. 3B) and Western blotting with anti-oligo-His antibodies (Fig. 3C) identified the ∼50 kDa species in the absence of reductant. This species was not found in the BioMNY sample containing Cys-less BioM, and disappeared in the samples containing mono-Cys BioM proteins upon treatment with 2-mercaptoethanol. Both the ∼55 kDa and ∼80 kDa species found in the membrane preparations (Fig. 3A) did not occur in case of the purified complexes shown in Fig. 3B and 3C. This finding reinforces our interpretation that the latter two bands represent a crosslinking product unrelated to BioMNY (∼55 kDa species) and a crosslinking product of BioM with an unrelated protein (∼80 kDa species). Thus, the data clearly indicate that all tested mono-Cys variants of BioM form homodimers by disulfide linkage.

### Mono-cysteine variants of BioN

**Figure 4:**
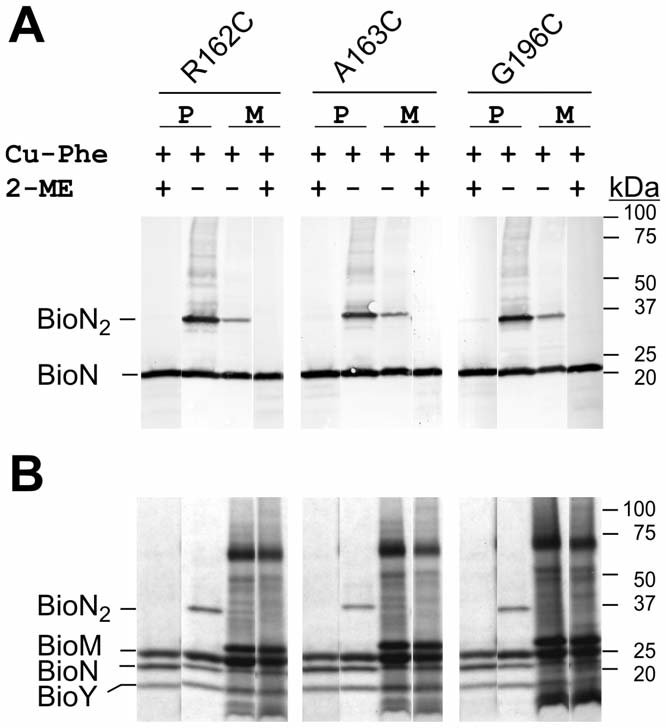
Crosslinking of selected c-Myc-tagged mono-cysteine BioN variants. Purified BioMNY complexes in detergent solution (*P*), or isolated membranes containing the complexes (*M*) were analyzed. *A.* Western blot with anti-c-Myc antibodies. *B.* Coomassie blue-stained gel.

**Figure 5:**
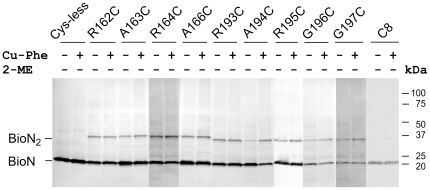
Electrophoretic mobility of mono-cysteine BioN variants. Isolated membranes containing BioMNY complexes with modified BioN peptides with or without Cu-phenanthroline (*Cu-Phe*) treatment were separated by SDS-PAGE, the gel was blotted onto a nitrocellulose membrane and the membrane was incubated with anti-c-Myc-tag antibodies.

A comparable strategy was applied for characterization of the nine variants of BioN containing a single Cys residue in or adjacent to the _163_ARS_165_ or _194_ARG_196_ motifs. First, BioMNY complexes containing the mutant BioN forms were purified and their ATPase activity was determined. The results are shown in Fig. S3 in the Supplement. They indicate that none of the replacements affected complex stability or abolished ATPase activity. In the next series of experiments, we investigated the sensitivity of the variants to thiol oxidation. BioMNY with the R162C, A163C and G196C BioN mutant proteins were randomly chosen and analyzed in the purified state in detergent solution and in isolated membranes. Fig. 4 illustrates the results. Unexpectedly, we observed a slower-migrating BioN form comigrating with ∼35 kDa in each case. The theoretical molecular mass of tagged monomeric BioN is 23.3 kDa. It comigrates with ∼21 kDa in SDS gradient gels. The appearence of the ∼35 kDa form in purified BioMNY complexes containing only a single cysteine residue (in BioN) and its disappearence upon addition of reductant strongly indicate that this species represents crosslinked BioN. Specific signals with the same electrophoretic mobility (∼21 kDa; ∼35 kDa) were also detected in all samples of isolated membranes, in which BioN is not a dominant peptide (Figs. 4 and 5), and even in the absence of Cu-Phe as the crosslinking agent. The spontaneous oxidation and the fact that only a single crosslinked product was observed with the membrane samples argue against random collisions as the basis of crosslinking. The formation of crosslinked BioN_2_ was not affected by lowering the temperature (by performing all steps on ice; data not shown). Upon addition of a reductant (dithiothreitol) to the isolated membranes, the dimeric BioN disappeared. It reappeared during incubation on ice in the presence of Cu-Phe within minutes (not shown) to give the pattern as presented in Fig. 5. Dimers were hardly visible in case of a BioN variant harboring its natural Cys residue (Cys8) (Fig. 5) in the N-terminal cytoplasmic loop which is predicted to be surface exposed. These findings support a view that BioN can dimerize specifically via the conserved ARS and ARG signatures. On the other hand, only a portion of the dimeric species was detectable for the BioN variants and the dimeric fractions could not be enhanced by prolonged incubation with Cu-Phe (not shown). The BioN variants with single cysteine residues in or adjacent to the _163_ARS_165_ motif on one hand, and the _194_ARG_196_ motif on the other hand gave oxidation products with slightly different electrophoretic mobility (Fig. 5), indicative of different conformations.

### Properties of BioMNY variants with mono-cysteine BioM plus mono-cysteine BioN

Sixtyfour BioMNY variants with mono-Cys BioN plus mono-Cys BioM were constructed and analyzed. The results of ATPase assays with the purified complexes are shown in Fig. S4 in the Supplement. Most of the double mutants had significant ATPase activity. The presence of the BioN_R164C_ form had led to low yields of BioMNY during purification and the complexes had very low ATPase activity. Low ATPase activity was an expected result since it was also observed for the BioN_R164C_/BioM_Cys-less_ pair (Fig. S3). In contrast, double mutants with the D86C replacement in BioM had high ATPase activity which may be due to the formation of functional disulfide-bridged BioM homodimers. Very low ATPase activity was found for double mutants with BioN_I98C_ and BioN_L102C_ and these variants gave rise to significant amounts of BioN-BioM heterodimers (see below).

### Site-specific crosslinking of BioN to BioM

**Figure 6:**
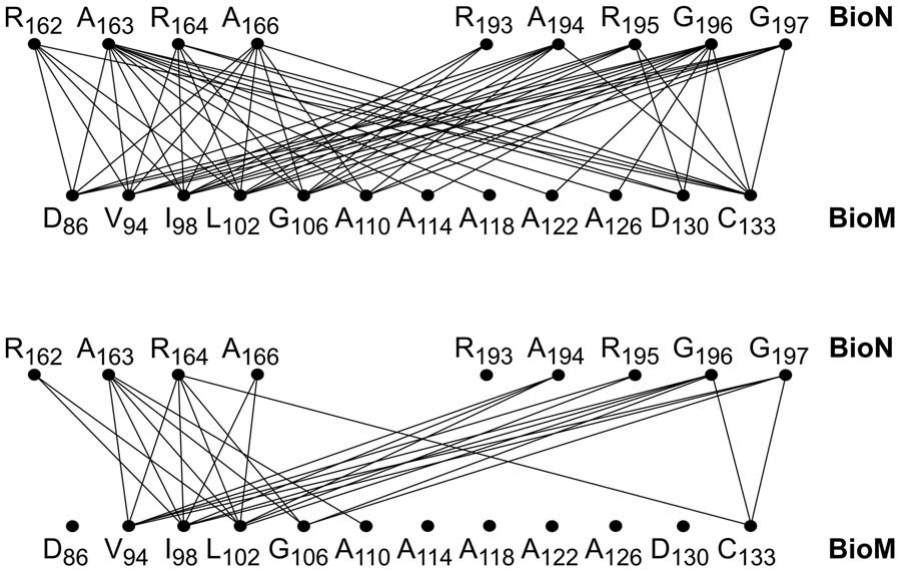
Overview of the mono-Cys BioN/mono-Cys-BioM pairs subjected to crosslinking. The *upper part* shows the 64 pairs analyzed and the *lower part* those 28 pairs giving strong signals for the BioN-BioM heterodimer.

**Figure 7:**
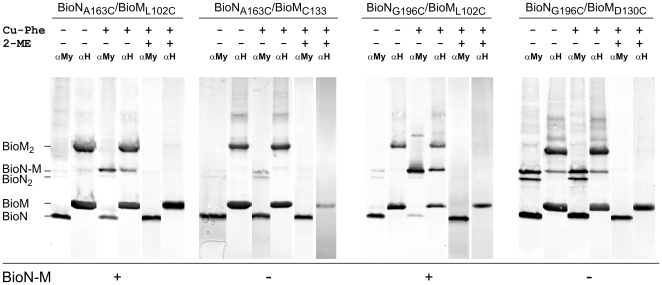
Crosslinking of BioMNY with mono-Cys BioN plus mono-Cys BioM in isolated membranes. Four selected BioN/BioM pairs are shown. Membranes were treated with Cu-phenanthroline (*Cu-Phe*) and 2-mercaptoethanol (*2-ME*) as indicated and subsequently solubilized with SDS-containing sample buffer. Proteins were blotted onto nitrocellulose membranes, and the membranes were treated with anti-oligo-His (*αH*) or anti-c-Myc (*αMy*) antibodies. The *plus* and *minus* below the line at the bottom of each panel refer to the occurrence of strong Cu-Phe-enhanced signals for the BioN-BioM pair. The crosslinking results of all 64 BioMNY variants with engineered mono-Cys BioN/mono-Cys BioM pairs are shown in Fig. S5 in the Supplement.

The 64 BioMNY variants with mono-Cys BioN plus mono-Cys BioM are schematically depicted in the upper panel of Fig. 6. The lower part of Fig. 6 indicates those 28 variants that gave a strong BioN-BioM crosslinking product. Fig. 7 illustrates exemplarily the crosslinking results for four BioN/BioM pairs. In the absence of reductant BioM homodimers were found in all mutant complexes, and in a number of complexes (e.g. in the variant with the BioN_G196C_/BioM_D130C_ pair, Fig. 7) significant amounts of homodimeric BioN was detected. Only those species were interpreted as specific BioN-BioM crosslink products that were significantly enriched upon treatment with Cu-Phe and reacted with both the anti-c-Myc (identifying the BioN moiety) and the anti-oligo-His (identifying the BioM moiety) antibodies. The complete data set for all 64 variants plus the BioN_C8_/BioM_V94C_ pair as a control is contained in Fig. S5 in the Supplement. Formation of BioM_2_ and BioN_2_ homodimers occurred spontaneously in many cases whereas addition of Cu-phenanthroline mostly enhanced crosslinking of BioN to BioM. All in all the results are in agreement with the aforementioned conclusion that BioN exists as a dimer (or oligomer) in BioMNY complexes. Specifically, the data show a narrow range of contact sites of the _163_ARS_165_ and _194_ARG_196_ regions in BioN with BioM residues V94 to G106 plus C133.

## Discussion

The molecular basis of physical and functional interactions among the subunits of ECF transporters is largely unknown. It has been proposed that an Ala-X_3_-Ala signature that is contained in the N-terminal part of eight S units of *L. lactis* and was shown by structure determination to be located within the first transmembrane helix of the S unit ThiT plays an important role in S unit:T unit interactions [14]. The role of the cytoplasmic Arg-containing motifs in a putative helical segment, a strongly conserved signature in T units, was the focus of the present study. We chose the BioMNY system for investigation because previous mutagenesis data had indicated that single replacements of the two Arg residues in BioN were tolerated to a certain extent. In contrast, subgroup II ECF transporters were completely inactivated by the corresponding exchanges in the T unit [12]. The cytoplasmic localization of the ARS and ARG signatures in BioN made them potential contact site(s) with the A unit(s) BioM.

As a precondition for site-specific cysteine crosslinking and immunological characterization, a Cys-free quadruple mutant of BioMNY was constructed and a c-Myc-tag was attached to BioN. The resulting variant complex retained significant ATPase activity and was used for construction of 64 pairs of mono-Cys BioN plus mono-Cys BioM double-mutant complexes. Two or three types of crosslinking products were obtained depending on the pairs analyzed. One species represents the dimeric BioM. The occurrence of this form was not surprising because dimers are the active form of well-characterized NBDs and recent in vivo analyses had confirmed an oligomeric state of BioM [11]. Additional evidence for dimerization of BioM involving the Q loop and the neighboring segment comes from the behaviour of the BioM_D86C_ variant. This species showed a strong homodimer signal (Fig. 3), it did not crosslink to any of the mono-Cys BioN variants (Fig. S5), it destabilized the BioMNY complexes (as indicated by partial loss of BioN and BioY during purification; Fig. S4), and it displayed significantly higher ATPase activity compared to other BioM forms (Fig. S4). The latter finding is compatible with the hypothesis, that dimerization via position 86 results in a BioM_2_ that is enzymatically active even in the absence of the transmembrane components.

The second type of crosslinking products represents homodimers of BioN. The formation of thiol-crosslinked homodimers of membrane proteins in vitro has been explained in different ways. It has been interpreted as consequence of (i) a naturally dimeric state (e.g. in the case of the YiiP heavy metal transporter [20]), (ii) random collisions within the membrane of monomeric species at room temperature (e.g. in the case of the lactose permease [21], [22]), and (iii) natural dimers, if some crosslinking occurs spontaneously and the crosslinking is not reduced by low temperature that diminishes lateral diffusion in the membrane (e.g. in the case of the potassium channel-like protein KtrB [23]). The crosslinking data in the present study show that all mono-Cys BioN and many variants with mono-Cys BioN plus mono-Cys BioM spontaneously form BioN homodimers, and that the apparent efficiency of this spontaneous or Cu-phenanthroline-catalyzed homodimerization is not affected by low temperature. Moreover, BioN peptides crosslinked specifically to homodimers in isolated membranes, although BioN did not represent the major protein species in these samples. On the other hand, under any conditions tested and even after prolonged incubation with Cu-phenanthroline, only portions of the BioN variants were found to dimerize. Thus, we consider a dimeric architecture of BioN in BioMNY complexes as a possibility but additional lines of evidence are required for proof.

**Figure 8:**
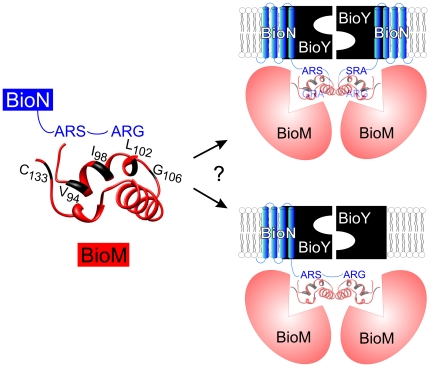
Model for BioMNY complexes. The two model variants in the right panel integrate data from previous analyses and from the present study. The substrate-specific component BioY was proposed to oligomerize in vivo [11]. BioM, a typical ABC ATPase, is predicted to function as a dimer and was found in an oligomeric state in BioMNY complexes in vivo [11]. Interactions between BioN and BioM via a cytoplasmic helical region of BioN, containing the ARS and ARG motifs, were proposed on the basis of physiological and biochemical analyses of mutant complexes [12]. Crosslinking analyses presented here detected physical contact sites between the ARS and ARG motifs in BioN and a stretch of residues adjacent to the Q loop in BioM as indicated in the zoom-out in the left panel. The BioM segment was structurally modelled using the SWISS-MODEL server at http://swissmodel.expasy.org. The present study identified specific crosslinks between pairs of BioN, a finding that may point to a dimeric architecture of this peptide in BioMNY complexes which is depicted in the upper right panel. The molecular basis of functional interaction between BioY and BioM in the absence of BioN [5] is unknown and not depicted in the two variants of the model.

The third type of crosslinking products represents BioN-BioM heterodimers. Among 64 pairs analyzed, we observed strong heterodimer signals in 28 cases. The results point to interactions of the ARS and ARG signatures in particular with a stretch of ∼13 residues (V94-G106) and with C133, which are located adjacent to the Q loop of BioM and in proximity to each other according to a structural model (Fig. 8). This segment is located at a similar position as the ENI motif in the small canonical ABC importers (like the maltose transporter) in which it contributes to coupling between the NBDs and TMDs [24].

Data presented here confirm our earlier experimental results and hypothesis [12] that the conserved Arg-containing signatures in a long cytoplasmic loop in T units are in physical contact with the A units in subgroup I and subgroup II ECF transporters. As predicted for the T unit of *L. lactis* [14] and shown in the present study for BioN, this helical part of the T proteins docks to the A unit(s) at a possible groove formed by residues located adjacent to the Q loop. Beyond the present study, the multitude of mono-Cys BioN and mono-Cys BioM variants and combined pairs derived thereof will allow more specific future experiments, e.g. crosslinking with bifunctional crosslinkers containing defined spacer lengths, and biophysical analysis using spin-labeled thiol-reactive agents.

The presently available data on the composition of BioMNY, the prototype of subclass I ECF transporters, were integrated to give two versions of a model illustrated in Fig. 8. The left part of the figure illustrates the interaction sites of the ARS and ARG signatures in BioN with defined positions in BioM. The oligomeric-state models in the right panel show BioY as a dimer. This reflects fluorescence analyses in living recombinant bacterial cells which uncovered specific FRET between BioY peptides indicating close spatial proximity [11]. It also agrees with recent unpublished results indicating that dimerization is essential for in vivo transport activity (F. Kirsch, T. Eitinger, unpublished result). Dimerization of BioM is conceivable on the basis of both theoretical considerations and experimental results of a previous [11] and the present study. A dimeric state of BioN is indicated as a possibility based on the experimental findings discussed above. The interactions of BioY with its partners remain elusive. Previous work has failed to identify stable BioNY and BioMY complexes (in the absence of the respective third partner) although transport studies suggest that labile but functional BioMY complexes may exist [5].

Additional biochemical and structural analyses are required to elucidate the in vivo oligomeric state of BioN. Moreover, it remains to be uncovered whether there is a common overall architecture of ECF transporters in vivo or subgroup I and subgroup II systems differ in oligomeric composition.

## Materials and Methods

### Recombinant bacterial strains

*Escherichia coli* XL1-Blue (Stratagene) was used for selection and maintenance of plasmid constructs. For purification of biotin transporter complexes, variants of plasmid pRc10HBioMNY encoding deca-His-tagged BioM, BioN, and FLAG-tagged BioY were introduced into *E. coli* UT5600, and the complexes were purified as previously described [5]. Construction of a derivative of this plasmid encoding a c-Myc epitope-tagged BioN is described below.

### Plasmid constructions

The deca-peptide EQKLISEEDL (c-Myc-tag)-encoding sequence was fused by inverse PCR to the 3′-end of *bioN* by the following procedure: Plasmid pRc10HBioMNY was used as the template and Phusion polymerase (New England Biolabs) for polymerization. The reverse primer consisted of the minus strand of a c-Myc-tag sequence containing a recognition site for the PpuMI restriction endonuclease at the 5′-end, and 18 nucleotides of the minus strand of *bioN* (complementary to codons 207–202). The forward primer consisted of a PpuMI recognition sequence, the stop codon of *bioN* and 21 nucleotides representing the 5′-end of *bioY*. This strategy is illustrated in Fig. S1 in the Supplement. The amplicon was treated with PpuMI, ligated and transformed into *E. coli* XL1-Blue (Stratagene). A 564 bp-NdeI/KasI fragment of the isolated plasmid comprising the 3′-end of *bioN* with the c-Myc-tag sequence and a major part of *bioY* was used to replace the corresponding segment of pRc10HBioMNY, and its nucleotide sequence was verified.

A variant plasmid encoding a cysteine-less BioMNY complex was constructed in a multi-step process. The wild-type system contains four Cys residues, three in BioM (at positions 133, 145 and 235) and one in BioN (at position 8), that were replaced by Ala residues (C133A, C145A, C8A) or deleted (ΔC235). Individual mutations were introduced as follows: Both the C133A and C145A replacements were obtained by two rounds of PCR using Platinum Pfx DNA polymerase (Invitrogen) and the wild-type plasmid as template. In the first round, a mutagenic forward primer, and a reverse primer complementary to the 3′-end of *bioM* were used. The resulting amplicons (approx. 300 bp) were purified and used as reverse primers in the second round of PCR together with a forward primer overlapping with the NcoI site at the 5′-end of *bioM*. The resulting 719-bp products were treated with NcoI and BamHI to give 468-bp fragments. Those fragments were used to individually replace the corresponding wild-type fragments yielding the C133A and C145A mutations. The ΔC235 mutation was constructed by insertion of a TGA stop codon after the Gly233 codon. This was achieved by PCR using the aforementioned *bioM* forward primer and a mutagenic reverse primer overlapping with an SphI site at the 5′-end of *bioN*. Treatment of the amplicon with BamHI and SphI resulted in a 240-bp fragment used for exchange. By this procedure the *bioM* reading frame was shortened by the terminal Ala234 and Cys235 codons of *bioM*, and the naturally overlapping *bioM* stop and *bioN* initiation sites were separated by two nucleotides. The SphI site overlapping with the *bioM* start codon was retained. Likewise, the C8A mutation in *bioN* was generated by a single round of PCR using a mutagenic forward primer overlapping with the SphI site and a reverse primer overlapping with the 3′-end of *bioN*. Treatment of the PCR product with SphI and MscI yielded a 304-bp fragment with the C8A replacement. The C133A/C145A double replacement was constructed by the above strategy for the two single mutations using a plasmid with the C145A codon exchange as the template. The ΔC235/C8A double mutant was obtained by inserting a 777-bp HincII/SphI fragment carrying the ΔC235 mutation into the digested plasmid with the C8A mutation. Finally, the plasmid encoding the cysteine-free biotin-transporter components was assembled based on the C133A/C145A and ΔC235/C8A precursors by inserting an 841-bp SapI/BamHI fragment of the former into the latter.

Fourteen variants with single-cysteine residues in the Q loop and the adjacent helical region (positions 82–133) of BioM, and nine variants with single cysteines around the conserved three-amino-acid motifs ARS (four mutants) and ARG (five mutants) in BioN were produced. The plasmid encoding the cysteine-less BioMNY was used as the template in the PCRs. Cysteine codons in *bioM* were generated by two rounds of PCR. A reverse primer overlapping with the BamHI site in the 3′-region of *bioM* and mutagenic forward primers were used in the first round. The resulting amplicons (113–256 bp) were used as primers in the second round together with the above primer overlapping with the NcoI site at the 5′-end of *bioM*. The 468-bp NcoI/BamHI fragments of the second-round amplicons contained the individual Cys codons. Two strategies were applied for construction of the single-cysteine BioN variants. Replacements around _163_ARS_165_, and the R193C and R195C replacements around _194_ARG_196_ were obtained in a single PCR with mutagenic forward primers overlapping the StuI (ARS region) or NdeI (R193C, R195C) sites in *bioN*, and a reverse primer overlapping the KasI site in the 3′-region of *bioY*. StuI/KasI and NdeI/KasI fragments, respectively, derived of the amplicons were used for the replacements in the plasmid vector. The remaining exchanges in the ARG region (A194C, G196C, G197C) were constructed by two rounds of PCR using mutagenic reverse primers and an upstream forward primer in the first round, and these amplicons plus the KasI-overlapping reverse primer in the second round. NdeI/KasI-digested PCR products were used for insertion into the vector plasmid.

A total of 64 BioMNY variants with a single-Cys residue in BioM and another single-Cys residue in BioN were constructed. For this purpose NcoI/BamHI fragments comprising the relevant region of *bioM* were inserted into plasmids containing solitary Cys codons in *bioN*.

### Site-specific cysteine crosslinking with purified membrane fractions

Total membranes of recombinant *E. coli* UT5600 containing BioMNY complexes with either a mono-cysteine BioM or a mono-cysteine BioN variant, or a mono-cysteine BioM plus a mono-cysteine BioN variant were isolated and subjected to thiol oxidation as follows: 100 ml cultures were grown overnight in Lysogeny broth supplemented with ampicillin (100 µg/ml) and isopropyl-*β*-D-thiogalactopyranoside (1 mM) at 37°C. Cells were harvested and washed with 35 mM sodium/potassium phosphate buffer (pH 7.0). The cell pellets were frozen in liquid nitrogen and stored at −80°C. Prior to the assays, the cell pellets were thawed and resuspended in 2 ml of wash buffer containing protease inhibitor cocktail (complete, EDTA-free; Roche) and DNase I according to the manufacturers' recommendation. Cells were disrupted by two passages through a French pressure cell. Total membranes were collected by ultracentrifugation at approx. 94,000 *g*, and homogenized in 50 mM Tris/HCl, 300 mM NaCl, 15% (vol./vol.) glycerol, 20 mM imidazole, pH 8.0 to give a protein concentration of 3 mg/ml. Protein was determined by a bicinchoninic acid kit (Pierce). Thiol oxidation was catalyzed in the presence of copper-phenanthroline complexes [25]. 120 µl of the membrane suspension was mixed with additional 48 µl of homogenization buffer and 48 µl of a freshly prepared copper-phenanthroline solution (15 mM CuSO_4_, 45 mM *1,10*-phenanthroline), and the mixtures were incubated for 20 min at room temperature (or on ice, if indicated). The reactions were stopped by adding 24 µl of 50 mM *N*-ethylmaleimide. After 5 min the samples were split and 40 µl of fourfold concentrated SDS sample buffer with and without 4% (vol./vol.) 2-mercaptoethanol (2-ME) was added to the divided samples. Specificity of thiol crosslinking was judged by comparison of the oxidized versus reduced sample pairs upon SDS PAGE. To estimate the amount of spontaneous crosslinking under air, an aliquot of the membrane preparations was treated with *N*-ethylmaleimide in the absence of copper-phenanthroline (Cu-Phe), and reductant-free SDS sample buffer was added. The triple samples (−Cu-Phe/−2-ME; +Cu-Phe/−2-ME; +Cu-Phe/+2-ME; about 15 µg of protein in each lane; 2-ME-containing and 2-ME-free samples on individual gels) were separated by SDS-PAGE using 4–20% precast gradient gels (BioRad). Proteins were blotted onto nitrocellulose membranes and the membranes were treated individually with the monoclonal antibody alkaline phosphatase conjugates anti-oligo-His and anti-c-Myc (Sigma), to detect His_10_-tagged BioM and c-Myc-tagged BioN, respectively.

### Crosslinking of purified BioMNY complexes

6 µg of purified protein (in 50 mM Tris/HCl, 300 mM NaCl, 15% (vol./vol.) glycerol, 0.05% (wt/vol.) *n*-dodecyl-*β*-D-maltoside, pH 7.5) was oxidized for 20 min at room temperature in the presence of 3 mM CuSO_4_ and 9 mM *1,10*-phenanthroline, the reactions were stopped by adding *N*-ethylmaleimide (5 mM final concentration), and SDS sample buffer was added. As in the case of the above mentioned protocol with membranes, triple samples representing the Cu-Phe-oxidized (+Cu-Phe/−2-ME), the reduced (+Cu-Phe/+2-ME) and the spontaneously oxidized (−Cu-Phe/−2-ME) forms were generated. Proteins in the samples were separated by SDS-PAGE. The gels were either stained with Coomassie Blue or blotted onto nitrocellulose membranes, and the latter were processed with either anti-oligo-His or anti-c-Myc alkaline phosphatase conjugates.

### ATPase activity

ATPase activity of purified BioMNY complexes was determined as previously described [5] by quantitating the rate of inorganic phosphate released from ATP. All ATPase activities are given as mean values. If more than two values were averaged, the standard deviations are indicated.

## Supporting Information

Figure S1 **Fusion of the c-Myc-tag-encoding sequence to the 3′-end of** ***bioN*** **.**
(PDF)

Figure S2 **Mono-cysteine BioM variants.**
(PDF)

Figure S3 **Mono-cysteine BioN variants.**
(PDF)

Figure S4 **BioMNY variants with mono-Cys BioN plus mono-Cys BioM.**
(PDF)

Figure S5 **Crosslinking of BioMNY with mono-Cys BioN plus mono-Cys BioM in isolated membranes.**
(PDF)
