# Supplementary material for: Interactions among the A and T Units of an ECF-Type Biotin Transporter Analyzed by Site-Specific Crosslinking
Source: PLoS One. 2011 Dec 27;6(12):e29087. doi: 10.1371/journal.pone.0029087 (PMC3246461; doi:10.1371/journal.pone.0029087)

Figure S1. Fusion of the c-Myc-tag-encoding sequence to the 3'-end of *bioN*. In the natural state, *bioN* and *bioY* overlap by 11 base pairs and a TTG serves as the *bioY* initiation codon. The latter has been replaced by an ATG in previous work (Hebbeln et al., 2007). To insert the c-Myc-tag-encoding sequence, the primers highlighted in yellow and a *bioMNY*-containing plasmid were used in an inverse PCR reaction. The resulting amplicon was treated with PpuMI (recognition site underlined) and ligated. A KasI/NdeI fragment was used to replace the corresponding fragment of the original plasmid. In the resulting construct, the sequences encoding tagged BioN and BioY overlap by an A nuceotide. The c-Myc-tag and its coding sequence are shown in red letters, the asterisks mark the stop codon of *bioN*/tagged *bioN*. BioN and BioY peptide sequences are highlighted in gray and olive, respectively.

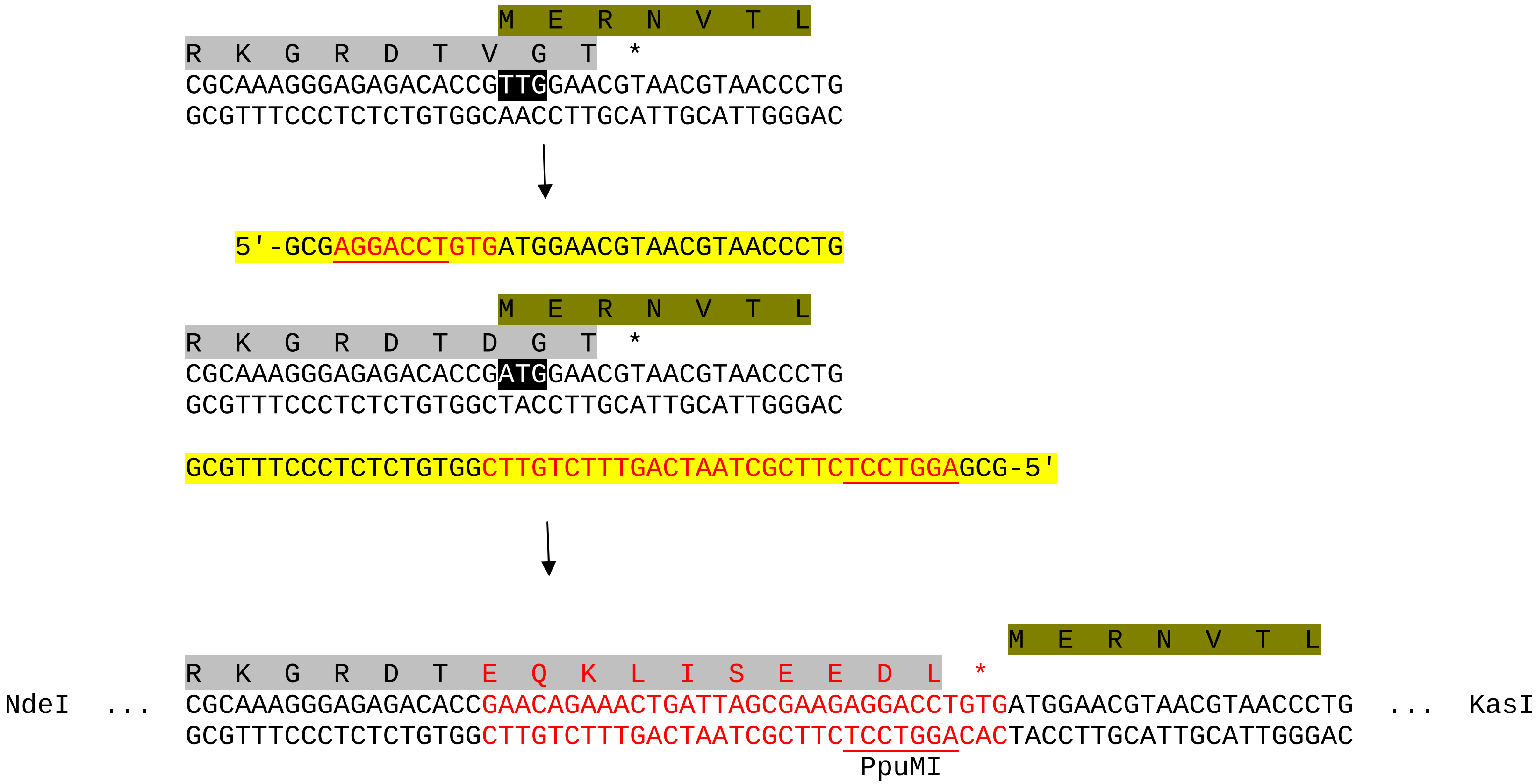

Supplement: Figure S1 — Fusion of the c-Myc-tag-encoding sequence to the 3′-end of bioN . (PDF) [file pone.0029087.s001.pdf]
