# Supplementary material for: Interactions among the A and T Units of an ECF-Type Biotin Transporter Analyzed by Site-Specific Crosslinking
Source: PLoS One. 2011 Dec 27;6(12):e29087. doi: 10.1371/journal.pone.0029087 (PMC3246461; doi:10.1371/journal.pone.0029087)

Figure S2. Mono-cysteine BioM variants. SDS-PAGE of purified BioMNY variants (approx. 2 μg of protein per lane) with mono-Cys BioM peptides. The experiments were done with BioMNY constructs lacking the cMyc-tag on BioN. Numbers below the samples give the ATPase activity in nmol P_i produced from ATP per min and mg of protein. *K42N* indicates a BioMNY variant with an inactivating exchange of the Walker A Lys residue of BioM.

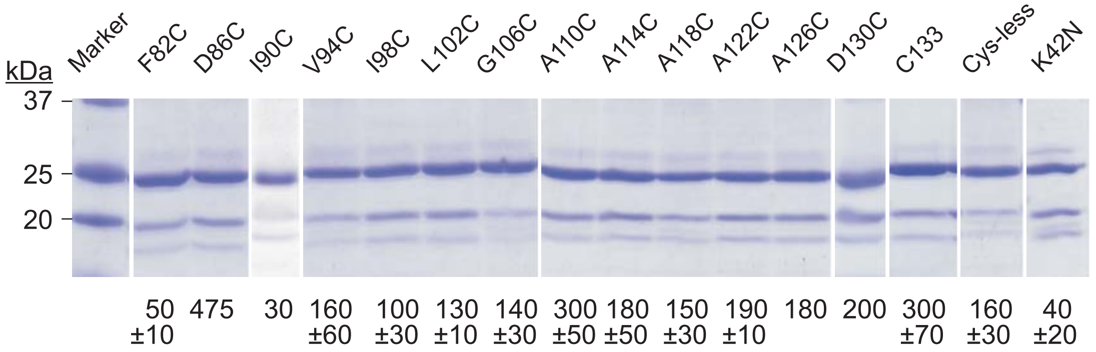

Supplement: Figure S2 — Mono-cysteine BioM variants. (PDF) [file pone.0029087.s002.pdf]
