# Supplementary material for: Interactions among the A and T Units of an ECF-Type Biotin Transporter Analyzed by Site-Specific Crosslinking
Source: PLoS One. 2011 Dec 27;6(12):e29087. doi: 10.1371/journal.pone.0029087 (PMC3246461; doi:10.1371/journal.pone.0029087)

Figure S3. Mono-cysteine BioN variants. SDS-PAGE of purified BioMNY variants (approx. 3 μg of protein per lane) with mono-Cys BioN peptides. Numbers below the samples give the ATPase activity in nmol P_i produced from ATP per min and mg of protein. *K42N* indicates a BioMNY variant with an inactivating exchange of the Walker A Lys residue of BioM.

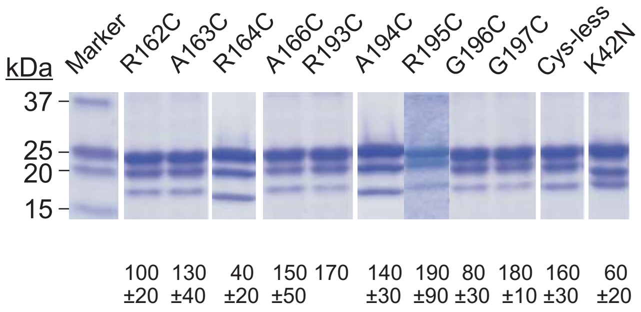

Supplement: Figure S3 — Mono-cysteine BioN variants. (PDF) [file pone.0029087.s003.pdf]
