# Supplementary material for: Interactions among the A and T Units of an ECF-Type Biotin Transporter Analyzed by Site-Specific Crosslinking
Source: PLoS One. 2011 Dec 27;6(12):e29087. doi: 10.1371/journal.pone.0029087 (PMC3246461; doi:10.1371/journal.pone.0029087)

Figure S4. BioMNY variants with mono-Cys BioN plus mono-Cys BioM. The result of SDS PAGE of purified complexes (approx. 3 μg of protein per lane) is shown. Numbers below the samples give the ATPase activity of the samples in nmol P_i produced from ATP per min and mg of protein.

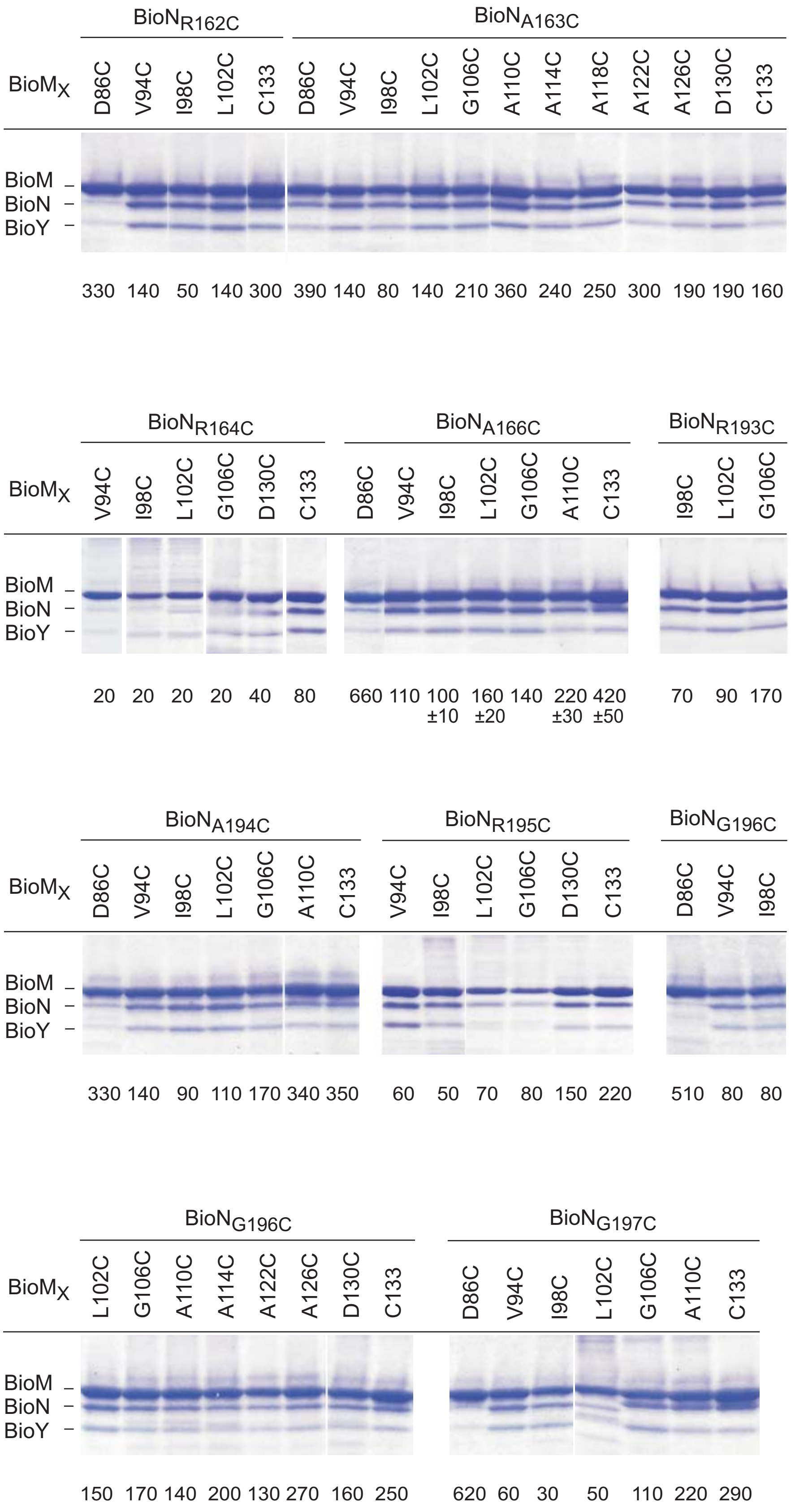

Supplement: Figure S4 — BioMNY variants with mono-Cys BioN plus mono-Cys BioM. (PDF) [file pone.0029087.s004.pdf]
