# Supplementary material for: Interactions among the A and T Units of an ECF-Type Biotin Transporter Analyzed by Site-Specific Crosslinking
Source: PLoS One. 2011 Dec 27;6(12):e29087. doi: 10.1371/journal.pone.0029087 (PMC3246461; doi:10.1371/journal.pone.0029087)
Supplement: Figure S5 — Crosslinking of BioMNY with mono-Cys BioN plus mono-Cys BioM in isolated membranes. (PDF) [file pone.0029087.s005.pdf]

Figure S5. Crosslinking of BioMNY with mono-Cys BioN plus mono-Cys BioM in isolated membranes. Membranes were treated with Cu-phenanthroline (*Cu-Phe*) and 2-mercaptoethanol (*2-ME*) as indicated and subsequently solubilized with SDS-containing sample buffer. Proteins were blotted onto nitrocellulose membranes, and the membranes were treated with anti-oligo-His (α*H*) or anti-c-Myc (α*My*) antibodies. The *plus* and *minus* below the line at the bottom of each panel refer to the occurrence of strong signals for the BioN-BioM pair upon addition of Cu-Phe.

$BioN_{R162C}/BioM_X$

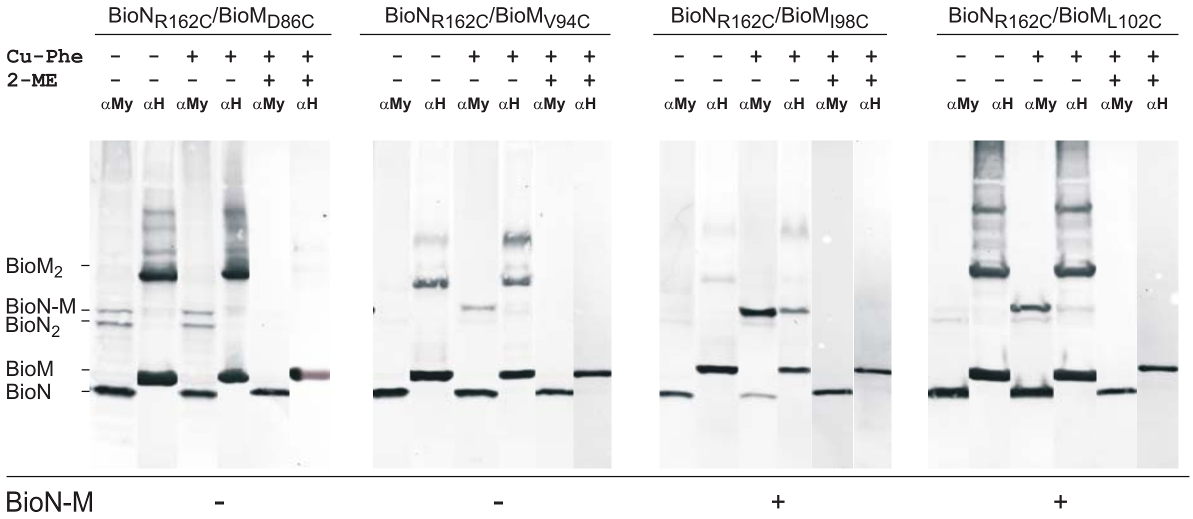

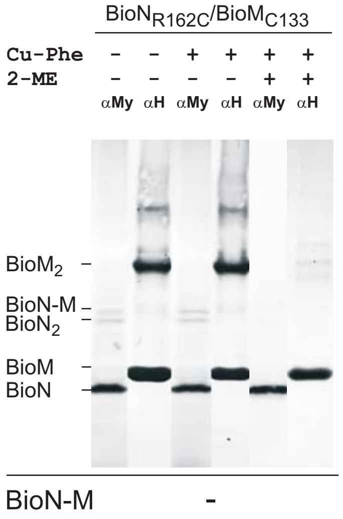

$BioN_{A163C}/BioM_X$

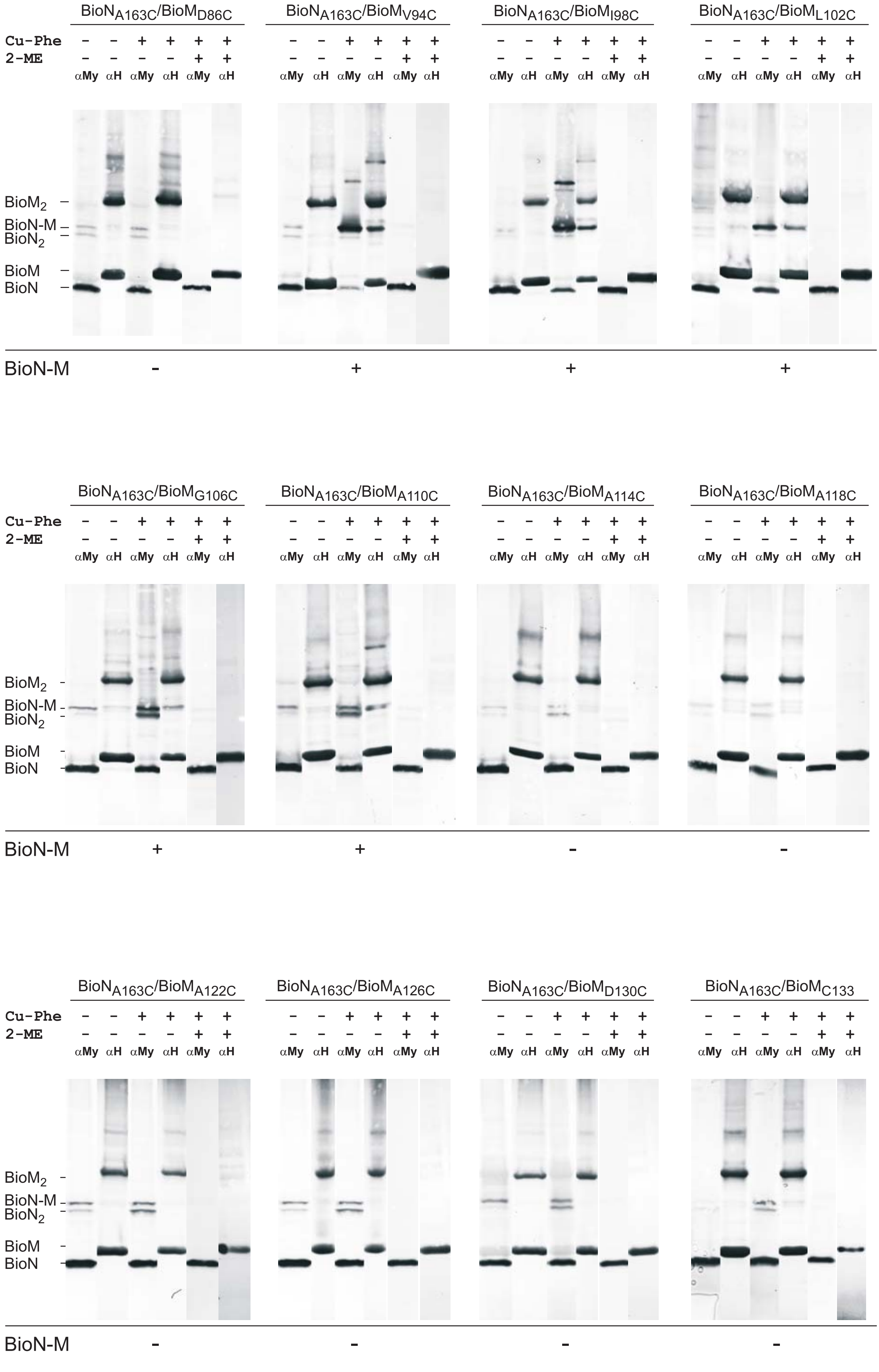

$BioN_{R164C}/BioM_X$

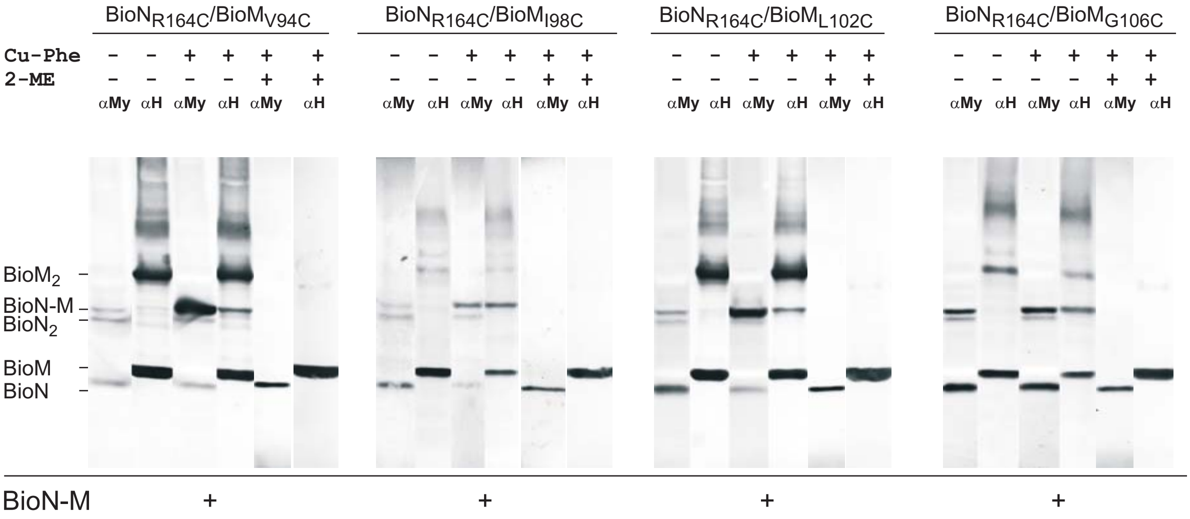

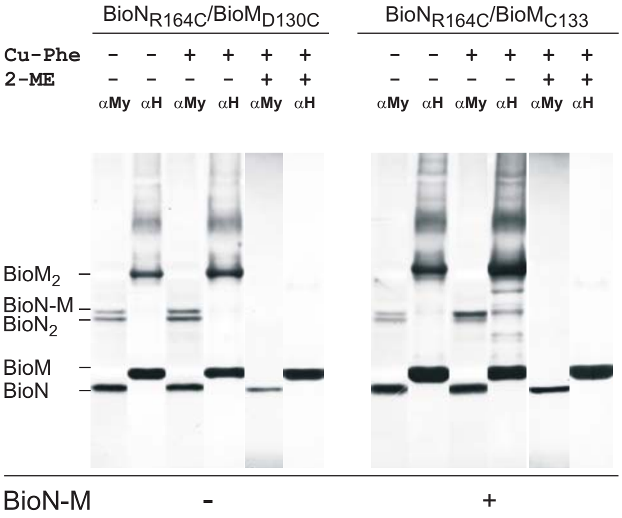

$BioN_{A166C}/BioM_X$

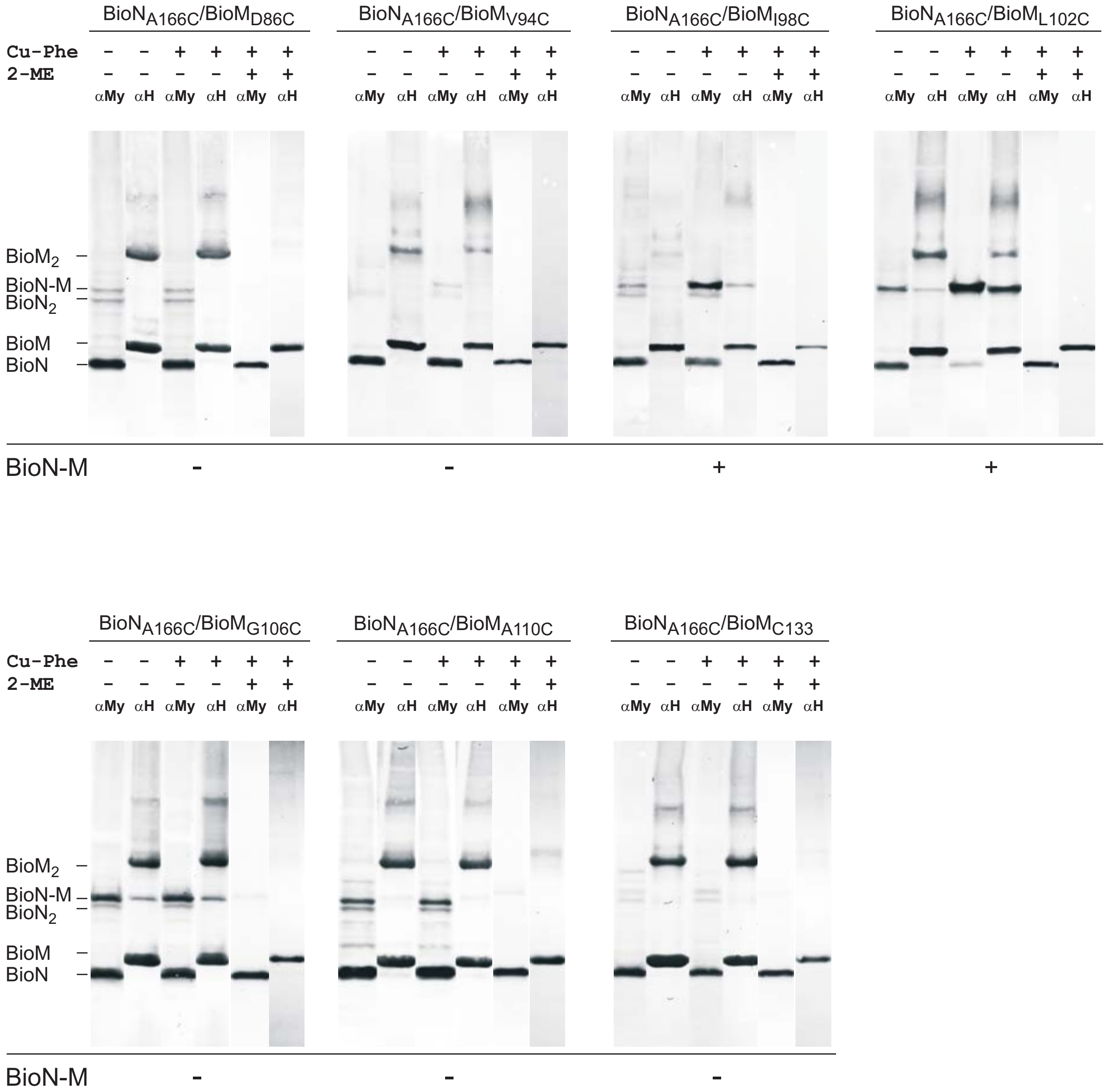

$BioN_{R193C}/BioM_X$

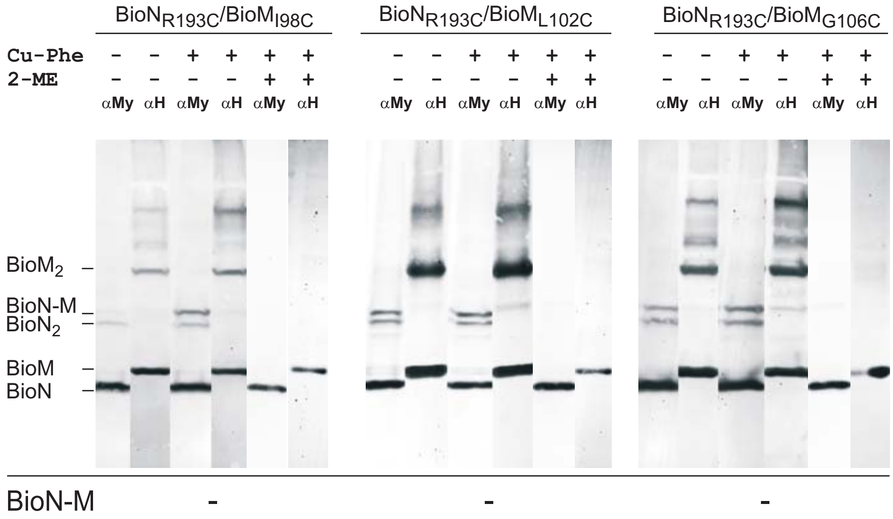

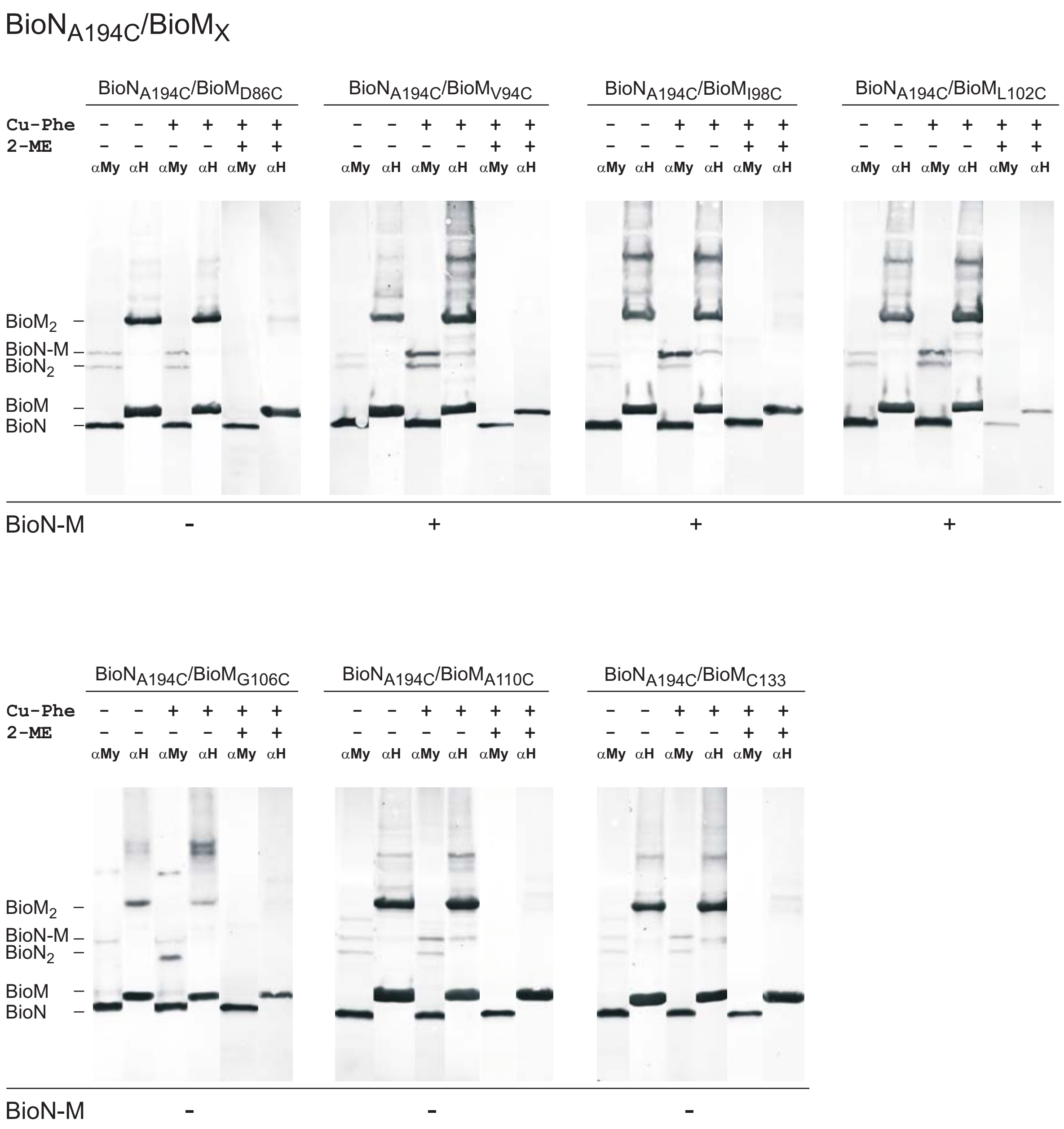
BioN$_{A194C}$/BioM$_X$
BioN$_{A194C}$/BioM$_{D86C}$
BioN$_{A194C}$/BioM$_{V94C}$
BioN$_{A194C}$/BioM$_{I98C}$
BioN$_{A194C}$/BioM$_{L102C}$
Cu-Phe
2-ME
αMy αH αMy αH αMy αH
BioM$_2$
BioN-M
BioN$_2$
BioM
BioN
BioN-M - + + +
BioN$_{A194C}$/BioM$_{G106C}$
BioN$_{A194C}$/BioM$_{A110C}$
BioN$_{A194C}$/BioM$_{C133}$
BioN-M - - -

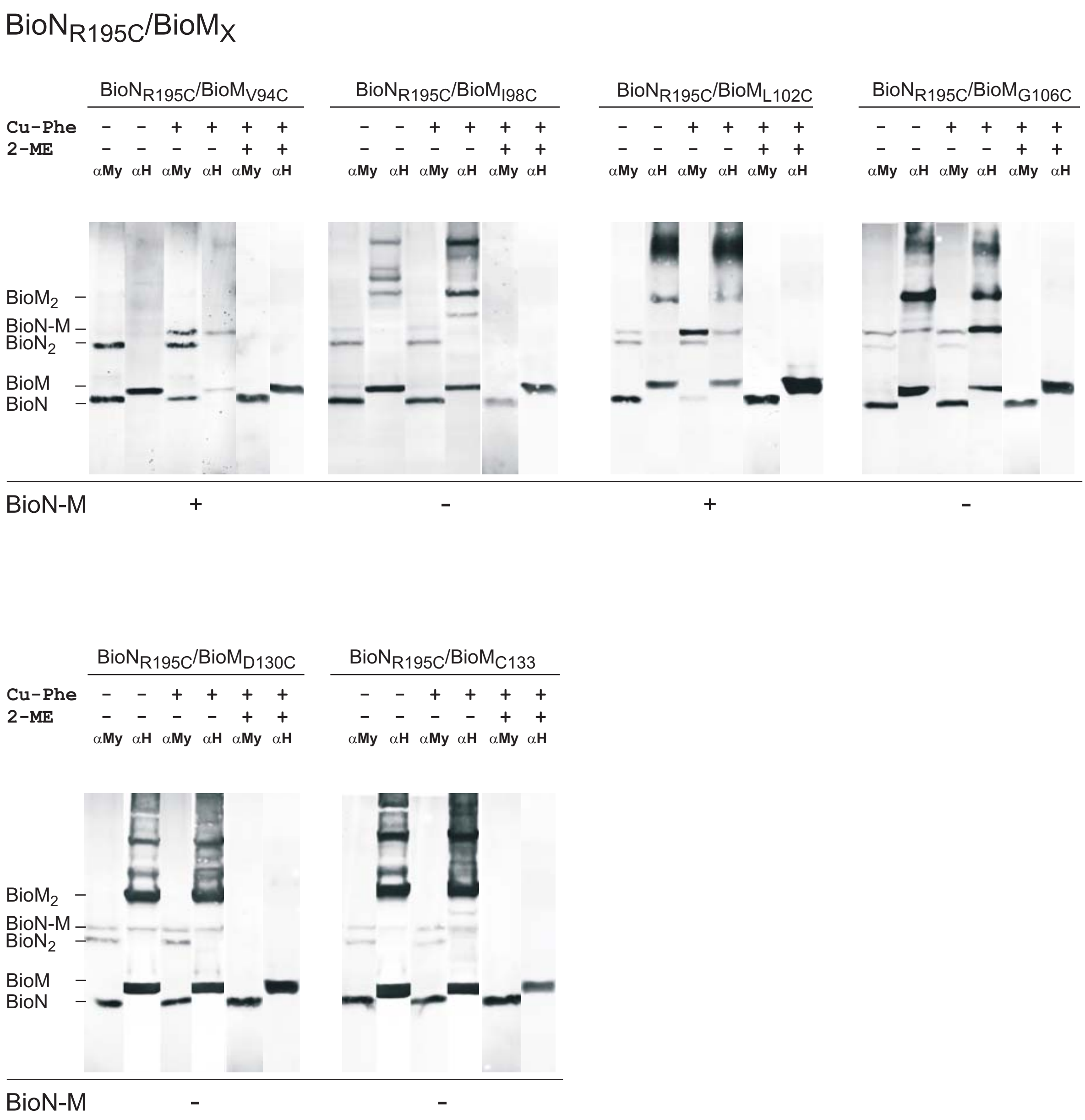

BioN$_{R195C}$/BioM$_X$
BioN$_{R195C}$/BioM$_{V94C}$
BioN$_{R195C}$/BioM$_{I98C}$
BioN$_{R195C}$/BioM$_{L102C}$
BioN$_{R195C}$/BioM$_{G106C}$
BioN$_{R195C}$/BioM$_{D130C}$
BioN$_{R195C}$/BioM$_{C133}$
Cu-Phe
2-ME
αMy
αH
BioM$_2$
BioN-M
BioN$_2$
BioM
BioN
BioN-M
+
-

$BioN_{G196C}/BioM_X$

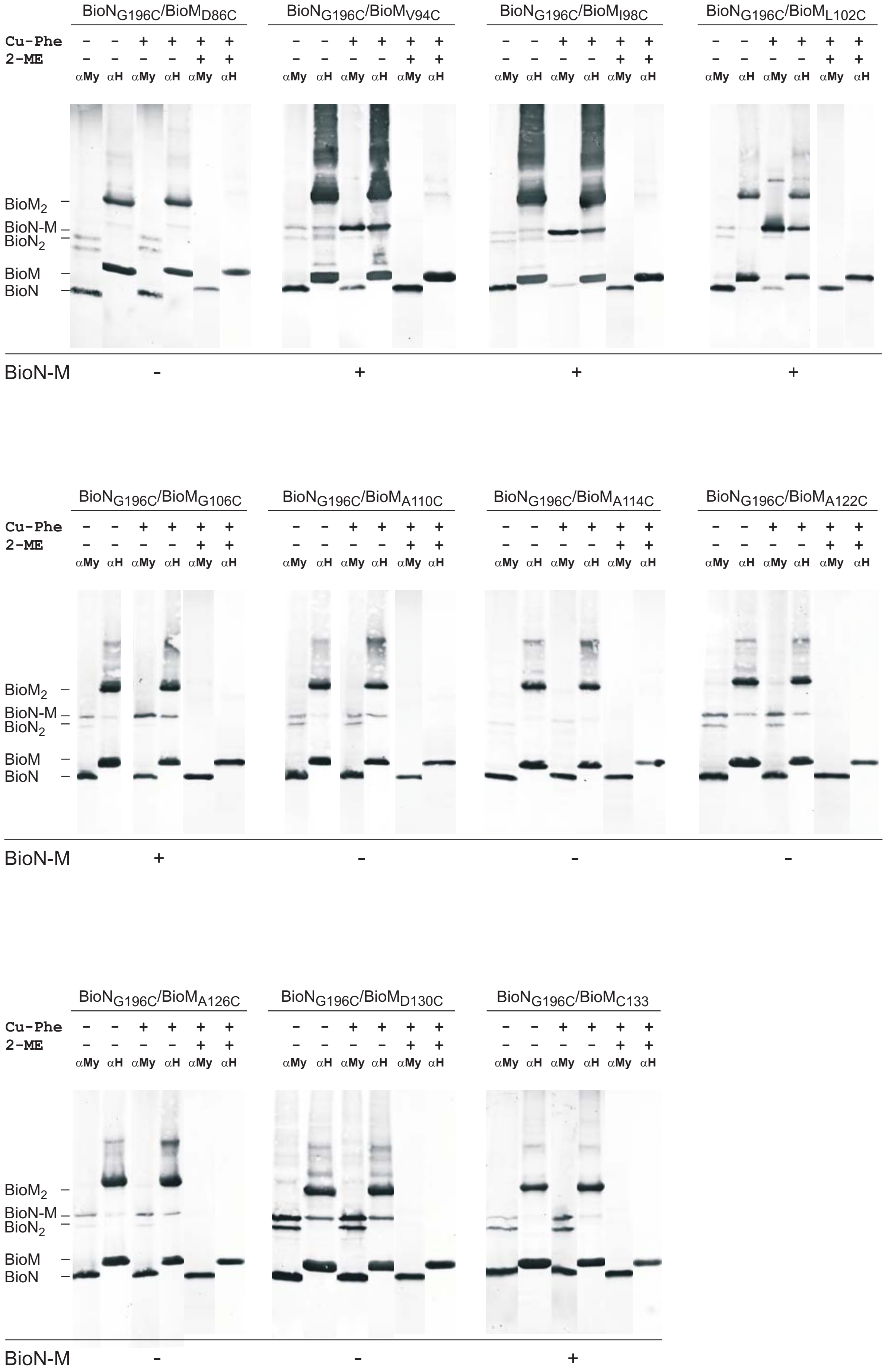

$BioN_{G197C}/BioM_X$

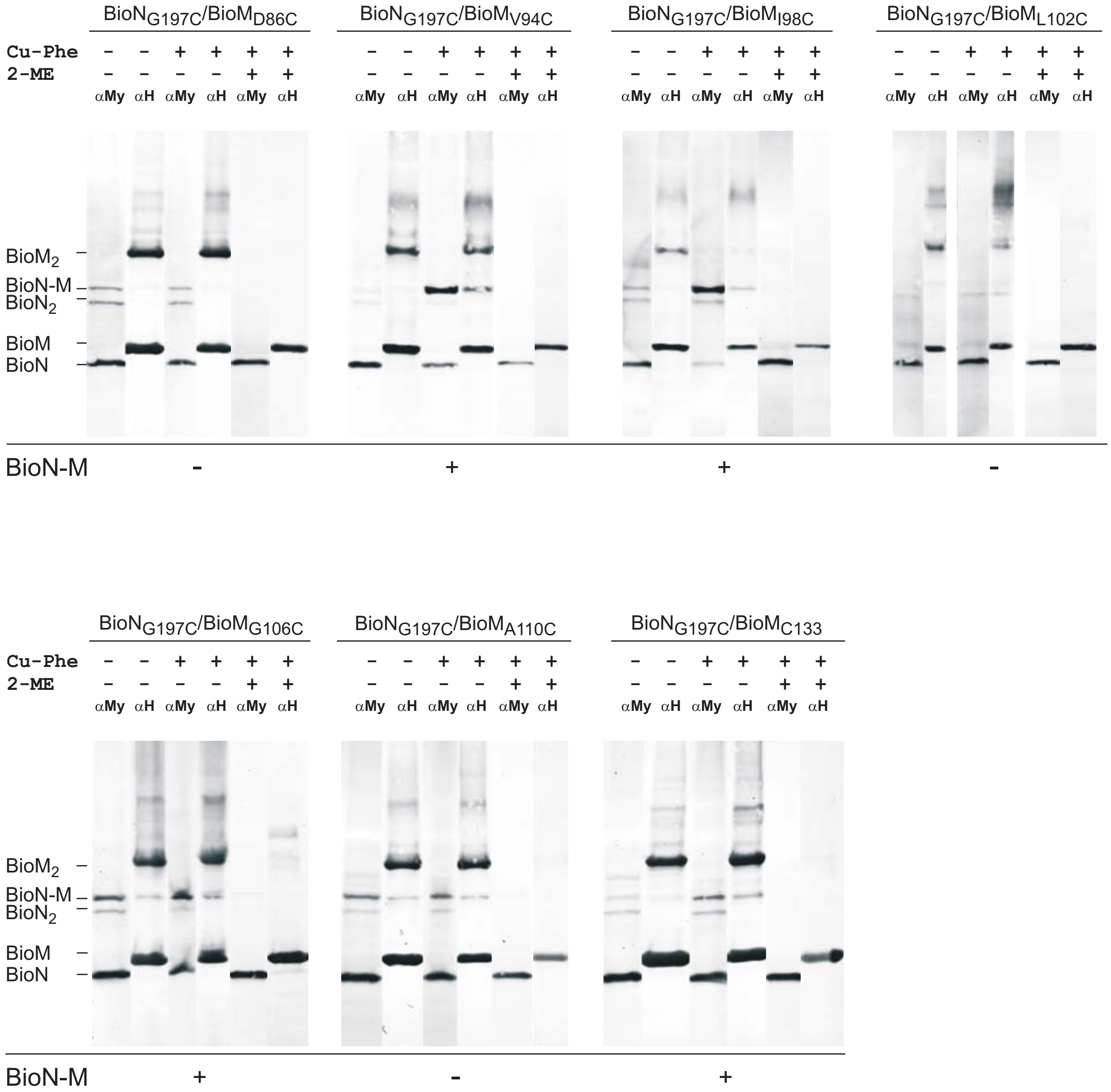

$BioN_{C8}/BioM_X$

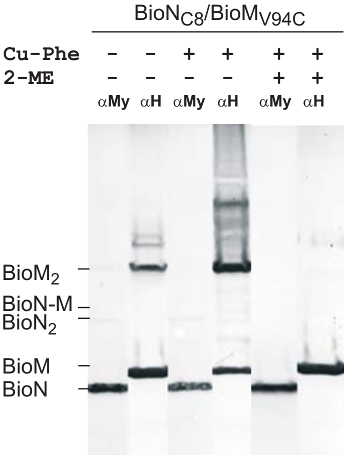

BioN-M -
